# Grain, Gluten, and Dietary Fiber Intake Influence Gut Microbial Diversity: Data from the Food and Microbiome Longitudinal Investigation

**DOI:** 10.1158/2767-9764.CRC-22-0154

**Published:** 2023-01-11

**Authors:** Caroline Y. Um, Brandilyn A. Peters, Hee Sun Choi, Paul Oberstein, Dia B. Beggs, Mykhaylo Usyk, Feng Wu, Richard B. Hayes, Susan M. Gapstur, Marjorie L. McCullough, Jiyoung Ahn

**Affiliations:** 1 Department of Population Science, American Cancer Society, Atlanta, Georgia.; 2 Department of Epidemiology and Population Health, Albert Einstein College of Medicine, Bronx, New York.; 3 Department of Population Health, New York University School of Medicine, New York, New York.; 4 New York University Perlmutter Cancer Center, New York, New York.; 5 Epidemiology Consultant, Tiffin, Iowa.

## Abstract

Although short-term feeding studies demonstrated effects of grains, fiber, and gluten on gut microbiome composition, the impact of habitual intake of these dietary factors is poorly understood. We examined whether habitual intakes of whole and refined grains, fiber, and gluten are associated with gut microbiota in a cross-sectional study. This study included 779 participants from the multi-ethnic Food and Microbiome Longitudinal Investigation study. Bacterial 16SV4 rRNA gene from baseline stool was amplified and sequenced using Illumina MiSeq. Read clustering and taxonomic assignment was performed using QIIME2. Usual dietary intake was assessed by a 137-item food frequency questionnaire. Association of diet with gut microbiota was assessed with respect to overall composition and specific taxon abundances. Whole grain intake was associated with overall composition, as measured by the Jensen–Shannon divergence (multivariable-adjusted *P*_trend_ for quartiles = 0.03). The highest intake quartile was associated with higher abundance of *Bacteroides plebeius*, *Faecalibacterium prausnitzii*, *Blautia producta*, and Erysipelotrichaceae and lower abundance of *Bacteroides uniformis*. These bacteria also varied by dietary fiber intake. Higher refined grain and gluten intake was associated with lower Shannon diversity (*P*_trend_ < 0.05). These findings suggest that whole grain and dietary fiber are associated with overall gut microbiome structure, largely fiber-fermenting microbiota. Higher refined grain and gluten intakes may be associated with lower microbial diversity.

**Significance:**

Regular consumption of whole grains and dietary fiber was associated with greater abundance of gut bacteria that may lower risk of colorectal cancer. Further research on the association of refined grains and gluten with gut microbial composition is needed to understand their roles in health and disease.

## Introduction

Whole grains are associated with lower risk of chronic diseases (1), including cancer and total cancer mortality, while refined grains do not have similar beneficial associations with cancer risk (2). Much of the evidence on cancer has focused on whole grains and colorectal cancer, and the World Cancer Research Fund/American Institute for Cancer Research (WCRF/AICR) has concluded that there is “probable” evidence that whole grains reduce colorectal cancer risk (3). As a result, the U.S. Dietary Guidelines to promote health and prevent chronic diseases recommend a healthy dietary pattern that includes grains, at least half of which are whole grains (4). Research on components of overall dietary patterns, such as of grains, helps to build evidence on the building blocks of healthy dietary patterns and inform research and public health messages on healthy diet.

The beneficial association between whole grains and colorectal cancer risk may be at least partly attributable to fermentation of these foods by short-chain fatty acid (SCFA)-producing gut microbes. Whole grains, unlike refined grains, are rich in fermentable dietary fiber and consequently have a “prebiotic” effect on gut microbes to produce SCFAs, such as butyrate, which are suggested to facilitate growth and differentiation of normal colonocytes while inhibiting tumor cell growth (5). Thus, diets rich in whole rather than refined grains are hypothesized to promote growth of SCFA-producing microbes while decreasing abundance of proinflammatory species (6, 7).

Gluten is another component of both whole and refined grains that may also alter gut microbiome composition. In the human gastrointestinal tract, gluten, composed of grain storage proteins, is cleaved into proline- and glutamine-rich peptides by proteolytic gut microbiota (8). Incomplete digestion of gluten can lead to immune responses that are characteristic of celiac disease, wheat allergy, and non-celiac gluten sensitivity. Although the role of gut microbiota is unclear in these conditions, differences in microbial composition have been observed in patients with celiac disease when compared with healthy populations (9), suggesting that certain microbes are needed in the digestion of gluten-containing foods.

Although strong evidence exists on the beneficial effect of whole grain foods on colorectal cancer risk, randomized controlled trials of whole grain foods and the gut microbiome have shown inconsistent findings (10). In addition, limited evidence from dietary intervention studies of gluten suggests that gluten-free or low-gluten diets alter gut microbial composition in healthy populations by decreasing several beneficial species associated with carbohydrate metabolism (11–13), but the evidence is largely based on small, short-term trials, similar to whole grain feeding studies (14–20). Little is known regarding the impact of habitual intake of these dietary factors on gut microbiota, and given that interest in gluten has steadily grown in recent years with increasing adherence to gluten-free diets (21), understanding the association of gluten-containing foods and gut microbiota is also of public interest.

To contribute to the limited evidence on gluten, grains, and gut microbiota, we examined self-reported habitual consumption of whole and refined grains, and their major components dietary fiber and gluten, with gut microbial composition, among participants of the Food and Microbiome Longitudinal Investigation (FAMiLI; ref. 22).

## Materials and Methods

### Study Population

The FAMiLI is an ongoing prospective cohort study of racially and ethnically diverse residents in New York City and surrounding geographic areas. Briefly, men and women ages 40 years or older who were not currently pregnant or on long-term antibiotic therapy were invited to enroll through clinic, community, and web-based recruitment. Participants with recent antibiotic use were eligible if the last dose was at least 2 weeks prior to the enrollment date. To date, over 9,500 participants have enrolled, with the goal of 15,000, and continued recruitment and prospective follow-up for disease outcomes are planned. At baseline, participants completed a questionnaire containing demographic, lifestyle, and dietary questions, and submitted self-collected oral and stool samples. The study was approved by the New York University Langone Health Institutional Review Board (#s12-00855), and all participants provided written informed consent. For the current analysis, participants who completed both demographic and dietary questionnaires and provided a stool sample were included, as published elsewhere (22). In the first phase, 1,000 participants were recruited and of those, 873 stool samples were selected for microbiome sequencing and included in this analysis.

### Dietary Assessment

Diet was assessed using a 137-item food frequency questionnaire (FFQ), known as the Dietary Questionnaire (DQX; ref. 23). The design of the DQX was based on two previously validated FFQs (24, 25) and is similar to the Diet History Questionnaire (DHQ), which was validated previously (26–29). The DQX was translated into Korean and Spanish for the FAMiLI participants. One additional page of culturally relevant foods was included with the DQX for Korean and Chinese participants, but because this page has not been validated, these food items were not included in the current study.

Study participants reported their frequency of consumption for 18 grain-containing items on the FFQ by selecting one of the 10 listed frequencies (never; less than once per month; one time per month; two to three times per month; one time per week; two times per week; three to four times per week; five to six times per week; one time per day; or two or more times per day) and one of the three listed serving sizes (small, medium, or large). The food items were categorized as whole or refined grains and as high- or low-gluten grain foods. Grain-based foods were classified as whole or refined based on the definition that whole grains retain 100% of the original kernel (30) and on extensive literature, including the WCRF/AICR systematic literature review on whole grains and colorectal cancer risk (3, 31). Whole grain foods in this analysis included high fiber cold cereals, dark wheat or rye breads, brown or wild rice, and other whole grains. Refined grain foods included cooked cereals or grits, other cold cereals, pancakes/waffles, white bread, corn bread, biscuits/muffins, white rice, pasta, pizza, and crackers. Five additional FFQ items were queried regarding refined grain-based sweets/dessert products; these items were not included in the primary analyses because they are frequently high in sugar and/or saturated fat, which may independently influence gut microbiota, but were included in sensitivity analyses.

To calculate the gluten content of whole and refined grain products, the amount of protein contained in wheat, barley, and rye food items was estimated from the USDA food composition database, FoodData Central (32). Then, the protein content was multiplied by a conversion factor of 0.75, based on the Osborne plant protein classification system (33). Previous estimates of the gluten fraction of proteins using this system applied conversion factors of 0.75 or 0.80 (34–36). Gluten estimation using the Osborne system has shown an acceptable correlation (*r* = 0.70; 95% confidence interval, 0.35–0.88) with gluten measurement using ELISAs (37).

The intakes of whole and refined grain, gluten, dietary fiber, and dietary fiber from grain-based foods were energy adjusted using the nutrient density method and then analyzed as continuous variables and sex-specific quartiles. For this analysis, participants who were missing ≥60 FFQ items, which also accounted for those missing >50% of the grain items (*n* = 48), and those who reported implausible total energy intake in the lowest or highest 1% (≤430 or ≥9,243 kcal/day for men and ≤412 or ≥6,703 kcal/day for women; *n* = 16) were excluded from this analysis. After exclusion of 64 subjects, 809 of 873 (93%) subjects remained.

### Gut Microbiome Assessment

Stool samples were self-collected by study participants upon enrollment using RNAlater collection kits. Participants were instructed to immediately ship the sample after collection, and average shipping time from the participant to the laboratory was 3–4 days. Because the samples were collected using RNAlater, shipments did not require cold or other special packaging. Samples were frozen immediately upon receipt until sequencing was performed. 16SV4 rRNA gene sequencing was conducted as described previously (22). DNA was extracted using the Mo Bio PowerSoil DNA isolation kit. The V4 region of the 16S rRNA gene was PCR amplified with the 515F/806R primer pair, which included sequencer adapter sequences used in the Illumina flowcell and sample-specific barcodes (38, 39). PCR products were quantified using PicoGreen (Invitrogen) and a plate reader (Infinite 200 PRO, Tecan). Sample PCR products were then pooled in equimolar amounts, purified using AMPure XP Beads (Beckman Coulter), and then quantified using a fluorometer (Qubit, Invitrogen). Amplicons were sequenced on a 151 bp × 12 bp × 151 bp MiSeq run (39).

Sequence reads were processed using QIIME2 (40). Briefly, sequence reads were demultiplexed and paired-end reads were joined, followed by quality filtering as described previously (41). The Deblur workflow was applied, which uses sequence error profiles to obtain putative error-free sequences, referred to as “sub” operational taxonomic units (s-OTU; ref. 42). s-OTUs were assigned taxonomy using a naïve Bayes classifier pretrained on the Greengenes (43) 13_8 99% OTUs, where the sequences have been trimmed to only include 250 bases from the 16S V4 region, bound by the 515F/806R primer pair. A phylogenetic tree was constructed via sequence alignment with MAFFT (44), filtering the alignment, and applying the FastTree algorithm (45) to generate the phylogenetic tree. The number of observed s-OTUs and Shannon diversity index were calculated in 100 iterations at 50 different rarefied sequencing depths (from 15 to 5,000 sequence reads per sample) and averaged for each subject at each depth (22).

In addition to the previously mentioned dietary exclusions, subjects for whom sequencing failed (*n* = 9) and subjects with sequencing depths <250 sequence reads per sample after the Deblur workflow (*n* = 21) were excluded, as described previously (22). After all exclusions, the final analytic cohort included 779 participants (291 men and 488 women). The data generated in this study are available upon request from the corresponding author.

### Statistical Analysis

The associations of sex-specific quartiles of energy-adjusted whole and refined grain, gluten, and dietary and grain fiber intake with within-subject microbial diversity (alpha-diversity) were evaluated using the number of observed s-OTUs (richness) and the Shannon diversity index. The dietary exposures were modeled using multivariable-adjusted linear regression models with richness and the Shannon index as outcomes, and adjusted for age, sex, race/ethnicity, body mass index (BMI; categories of 18–<25 kg/m^2^, 25–<30 kg/m^2^, 30–<35 kg/m^2^, ≥35 kg/m^2^), smoking status (ever/never), alcohol intake (g/day), and total energy intake (kcal/day, continuous). We also tested whole grain models additionally adjusted for refined grains and vice versa, and whole and refined grain and gluten models additionally adjusted for dietary fiber and fruit and vegetable (servings/day) intake. Trend tests across quartiles of intake were calculated using the median quartile values, modeled as a continuous term. The association of quartiles of whole and refined grain, gluten, and dietary and grain fiber intake with overall gut microbiota composition was assessed using Jensen–Shannon divergence (JSD). Permutational multivariate analysis of variance was performed using R software (version 3.6.1; R Development Core Team, 2020), R package “vegan” (46), and adonis function to assess this association (47), and models were adjusted for the same covariates mentioned previously.

The analysis of composition of microbiomes (ANCOM) method (48) was used to identify s-OTUs and higher level taxa that were differentially abundant between the highest and lowest intakes of the various dietary exposures, adjusting for the previously mentioned covariates. For this analysis, taxa at the detection level ≥0.80 were considered significant.

In sensitivity analyses, analyses were repeated stratified by racial/ethnic group and after excluding participants who self-reported type 2 diabetes (*n* = 130) or inflammatory bowel disease (Crohn’s disease or ulcerative colitis; *n* = 21) at enrollment. Grain-based sweets and desserts were also included in the estimation of refined grain and gluten intake. Analyses were performed using R version 3.6.1 (R Core Team, 2020).

### Data Availability

The 16S rRNA sequencing data that support the findings of this study have been deposited in the Sequence Read Archive (PRJNA559143), along with demographic metadata, to be released upon publication. Additional data on the study participants are available from the corresponding author upon reasonable request.

## Results

### Participant Characteristics

In this study population, 36.7% of participants were White, 10.4% were Black, 15.0% were Hispanic, 30.2% were Korean, 3.1% were other Asian, and 1.0% were other race/ethnicities. Participants with the highest intakes of whole grain were older, had a lower BMI, and had higher total energy, dietary fiber, and fruit and vegetable intakes but lower calories from fat and intakes of alcohol, processed meat, and refined grains (Table 1). In addition, greater whole grain consumers were more likely to be Korean, more highly educated, regular aspirin users, and physically active. Conversely, greater refined grain consumers were generally younger, heavier, less physically active, and had lower intakes of dietary fiber and fruits and vegetables.

**TABLE 1:** Baseline characteristics of study participants by sex-specific quartiles of energy-adjusted whole and refined grain intake in the FAMiLI

	Whole grain quartiles^a^^,^^b^				Refined grain quartiles^a^^,^^b^			
	Q1	Q2	Q3	Q4	Q1	Q2	Q3	Q4
	*N* = 196	*N* = 195	*N* = 193	*N* = 196	*N* = 195	*N* = 196	*N* = 193	*N* = 196
Range: Males	0–8.0	>8.0–20.2	>20.2–46.5	>46.5–299	0–47.7	>47.7–80.5	>80.5–120	>120–343
Females	0–10.3	>10.3–27.5	>27.5–63.8	>63.8–448	0–41.9	>41.9–74.4	>74.4–119	>119–370
Age (years)	57.6 (11.2)	57.2 (10.5)	60.2 (11.4)	62.8 (11.0)	64.5 (10.9)	59.7 (10.7)	58.2 (10.2)	55.4 (11.1)
Female, %	62.6	62.6	62.9	62.6	62.6	62.6	62.9	62.6
BMI (kg/m^2^)	29.1 (7.0)	28.5 (6.3)	27.6 (5.9)	24.4 (4.9)	25.9 (5.3)	27.5 (6.8)	28.4 (6.3)	27.9 (6.5)
Race/ethnicity, %								
White	37.9	52.3	48.5	13.8	26.7	43.1	44.8	37.9
Black	15.4	12.3	9.8	5.6	5.1	14.9	12.9	10.3
Hispanic	28.2	18.5	13.4	2.1	7.7	11.3	19.6	23.6
Korean	14.4	11.3	23.7	75.9	59.0	25.6	19.6	21.0
Asian, other than Korean	3.6	3.6	3.6	2.1	1.0	4.1	2.1	5.6
Other^c^	0.5	2.1	1.0	0.5	0.5	1.0	1.0	1.5
College graduate or higher, %	44.1	59.5	64.9	53.3	49.7	61.5	58.8	51.8
Physical activity, %								
None	21.0	13.3	7.7	11.8	9.2	10.8	13.4	20.5
<4 hours/week	56.4	60.0	54.1	49.2	44.1	61.0	62.4	52.3
4 or more hours/week	22.6	26.7	38.1	39.0	46.7	28.2	24.2	27.2
Ever smoker, %	33.3	41.0	31.4	24.6	30.8	36.9	30.9	31.8
Alcohol consumption (g/day)	8.0 (22.2)	14.2 (31.9)	8.0 (22.5)	4.0 (15.6)	9.4 (29.5)	9.6 (23.7)	9.6 (24.2)	5.6 (16.8)
Family history of cancer, %	55.9	55.9	53.1	39.0	45.6	57.9	53.1	47.2
Regular aspirin use, %	32.3	33.3	33.5	41.5	34.9	34.4	36.6	34.9
History of ulcerative colitis, %	3.1	1.0	2.1	2.6	1.5	2.6	2.6	2.1
History of Crohn’s disease, %	0.5	1.0	0.5	0.0	0.0	1.0	0.5	0.5
Total energy (kcal/day)	1,889 (1,047)	2,015 (1,212)	2,052 (1,050)	2,122 (1,036)	2,106 (1,017)	1,905 (935)	2,059 (1,064)	2,007 (1,306)
Total fat (% kcal/day)	31.2 (8.2)	30.9 (6.6)	29.4 (5.4)	25.8 (4.6)	28.0 (7.7)	29.9 (7.0)	30.2 (6.2)	29.2 (5.6)
Total carbohydrates (% kcal/day)	51.7 (11.2)	50.4 (9.4)	54.1 (8.1)	58.3 (7.0)	54.3 (11.2)	53.0 (10.3)	52.6 (8.3)	54.5 (7.9)
Red meat (servings/day)	0.6 (0.7)	0.7 (0.8)	0.6 (0.6)	0.5 (0.7)	0.6 (0.8)	0.5 (0.5)	0.6 (0.7)	0.6 (0.9)
Processed meat (servings/day)	0.7 (1.0)	0.6 (0.8)	0.4 (0.7)	0.2 (0.5)	0.4 (0.8)	0.5 (0.8)	0.6 (0.8)	0.5 (0.8)
Fruits and vegetables (servings/day)	5.9 (4.6)	7.2 (4.5)	8.1 (4.7)	9.6 (5.3)	9.8 (5.9)	7.2 (4.4)	7.4 (4.5)	6.4 (4.2)
Total dairy (servings/day)	2.1 (2.2)	2.2 (2.0)	2.0 (2.0)	1.3 (1.5)	1.7 (1.9)	2.0 (1.9)	2.2 (2.2)	1.7 (1.8)
Whole grains (energy-adj; g/day)	3.5 (3.1)	16.4 (5.0)	38.1 (10.2)	126.6 (74.9)	79.0 (88.5)	41.3 (51.6)	33.6 (38.4)	30.7 (39.3)
Refined grains (energy-adj; g/day)	105.2 (76.3)	98.0 (58.8)	92.9 (58.7)	62.8 (55.7)	23.1 (13.9)	60.9 (9.8)	95.2 (12.0)	179.7 (54.6)
Gluten (energy-adj; g/day),	2.1 (1.4)	2.7 (1.3)	3.2 (1.8)	2.8 (2.1)	1.6 (1.5)	2.3 (1.2)	3.0 (1.3)	3.9 (1.9)
Dietary fiber (energy-adj; g/day)	18.0 (10.2)	22.9 (13.2)	28.1 (15.8)	35.1 (17.8)	31.6 (18.3)	23.2 (13.0)	25.2 (14.0)	24.1 (16.3)

Abbreviations: BMI, body mass index; energy-adj, energy-adjusted.

^a^Whole and refined grain quartiles are energy-adjusted using the nutrient density method.

^b^Values presented are mean (SD) unless otherwise indicated.

^c^“Other” race/ethnicity included participants who reported more than one or mixed race/ethnicity.

### Whole Grain and Fiber Intake

**FIGURE 1:**
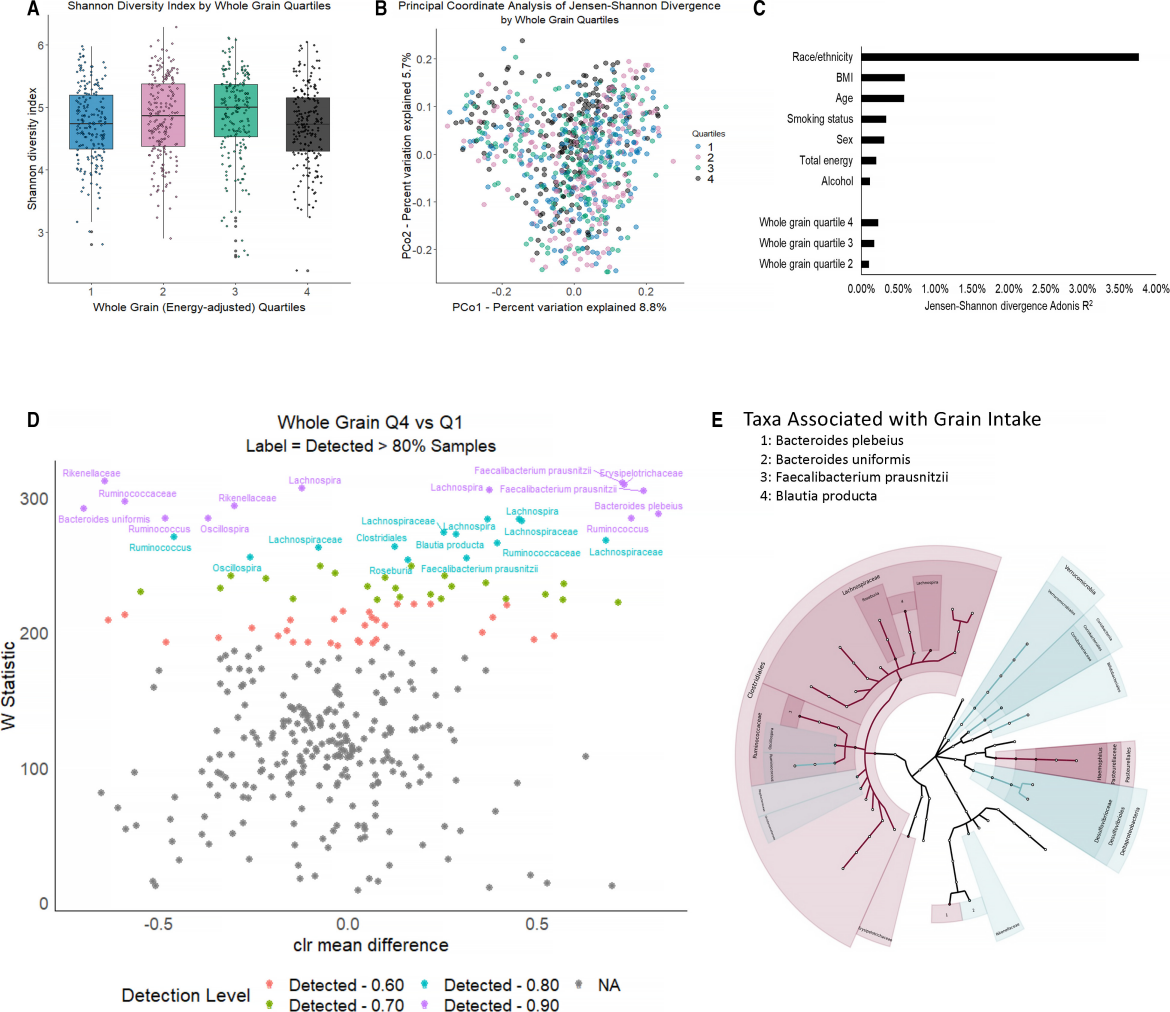
Gut microbiome alpha- and beta-diversity according to energy-adjusted quartiles of whole grain intake in the FAMiLI. **A,** Boxplot of Shannon diversity index by whole grain quartiles. **B,** Principal coordinate analysis of the JSD by whole grain quartiles. **C,** Bar plot illustrating the R^2^ for model covariates derived from JSD. **D,** Volcano plots of differentially abundant s-OTUs as detected by ANCOM (model adjusted for age, sex, race, BMI, smoking status, alcohol intake, and total energy) between quartile 4 (Q4) and quartile 1 (Q1) of whole grain intake. The *x*-axis represents the difference in mean centered log ratio (clr)-transformed abundance between Q4 and Q1, and the *y*-axis represents the ANCOM W Statistic. s-OTU points are colored according to level of ANCOM significance, with 0.90 being the highest level and grey points indicating s-OTUs that were not significant. **E,** Cladograms of phylum through species level taxa; color represents clr mean difference between Q4 and Q1 of whole grain intake.

Greater whole grain consumption was not associated with the number of observed s-OTUs (richness; *P*_trend_ = 0.96) or the Shannon diversity index (*P*_trend_ = 0.79; Fig. 1A; alpha-diversity) after adjustment for age, sex, race/ethnicity, BMI, smoking status, alcohol intake, and total energy intake. However, higher whole grain intake was associated with overall gut microbiome composition, as measured by JSD (Fig. 1B; beta-diversity, Q4 vs. Q1 R^2^ = 0.23%, *P* = 0.01; R^2^ for trend = 0.22%, *P*_trend_ = 0.02), with adjustment for age, sex, and race/ethnicity. The amount of variation remained consistent after further adjustment for BMI, smoking status, alcohol intake, and total energy intake (Q4 vs. Q1 R^2^ = 0.23%, *P* = 0.01; R^2^ for trend = 0.22%, *P*_trend_ = 0.02; Fig. 1C). We identified bacterial taxa associated with greater whole grain consumption (Fig. 1D): s-OTUs of *Bacteroides plebeius*, *Faecalibacterium prausnitzii*, and *Blautia producta* were enriched, while *B. uniformis* was depleted. Additional significantly differentially abundant s-OTUs belonged to the following higher-order groups: Erysipelotrichaceae and Roseburia were enriched at detection levels of 0.90 and 0.80, respectively, Oscillospira and Rikenellaceae were depleted, and Lachnospira, Ruminococcus, and Ruminococcaceae were both enriched and depleted. Dietary fiber, a major whole grain nutrient, was positively correlated with whole grain intake (*r* = 0.42) and was similarly associated with beta-diversity (JSD, Q4 vs. Q1 R^2^ = 0.21%, *P* = 0.02; R^2^ for trend = 0.20%, *P*_trend_ = 0.03) but not with alpha-diversity (*P*_trend_ for richness = 0.30, *P*_trend_ for Shannon diversity index = 0.38; Supplementary Fig. S1). Abundant taxa associated with greater dietary fiber intake (Supplementary Fig. S1C) were similar to those associated with whole grains (Fig. 1E). When whole grain models were additionally adjusted for dietary fiber or fruit and vegetable intake, the results for alpha-diversity (*P*_trend_ for richness = 0.59 and 0.84, respectively; *P*_trend_ for Shannon diversity index = 0.43 and 0.60, respectively) and for differentially abundant taxa did not materially change; however, whole grain intake was no longer significantly associated with microbial community diversity as measured by JSD after adjustment for dietary fiber or fruit and vegetable intake (R^2^ for trend = 0.19% and 0.15%, respectively; *P*_trend_ = 0.06 and 0.21, respectively). Grain fiber was similarly positively correlated with whole grain intake (*r* = 0.48), but higher intake was not significantly associated with alpha- (*P*_trend_ for richness = 0.54, *P*_trend_ for Shannon diversity index = 0.53) or beta-diversity (R^2^ for trend = 0.15%, *P*_trend_ = 0.19) or any specific microbial taxa.

### Refined Grain and Gluten Intake

**FIGURE 2:**
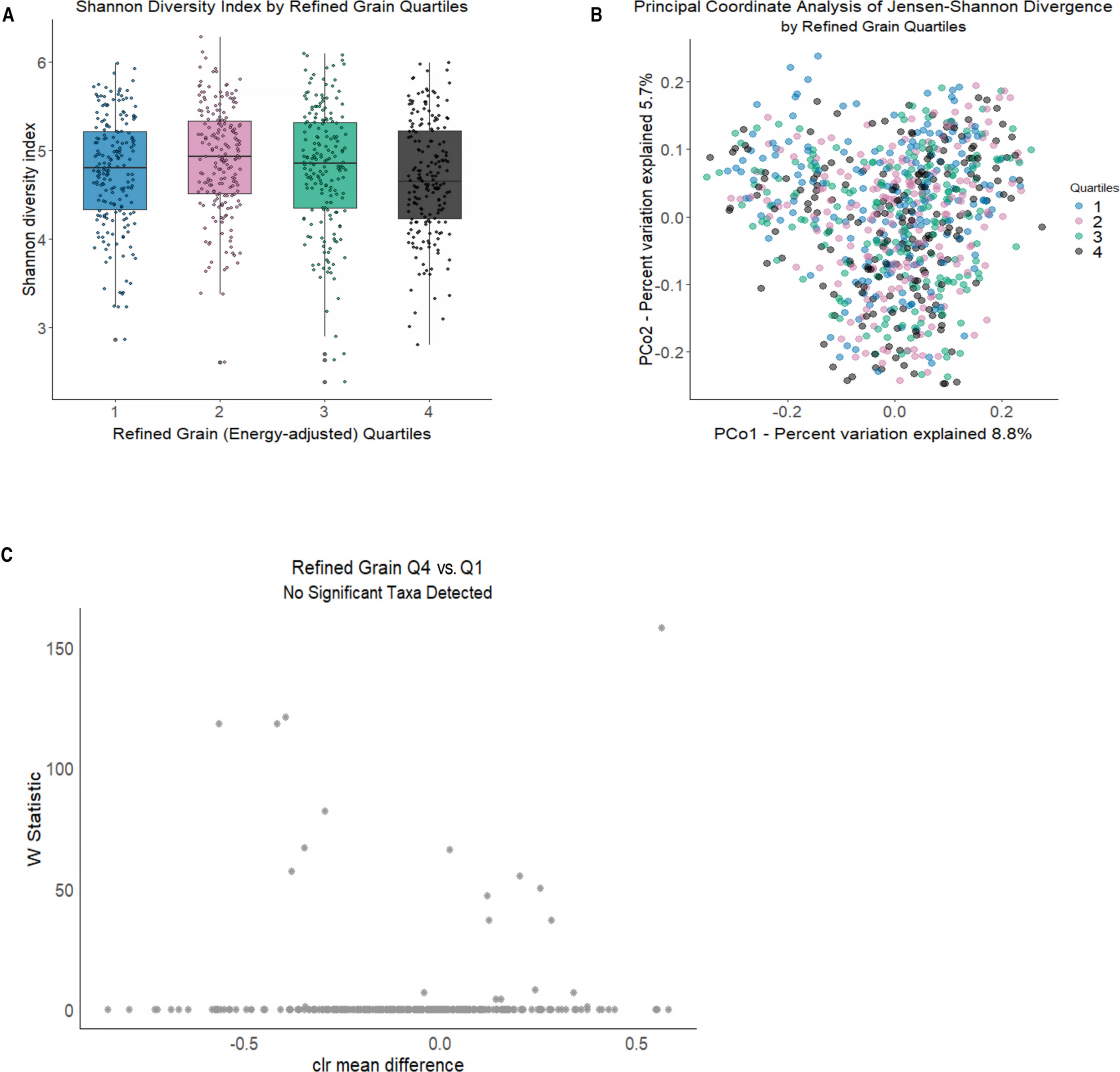
Gut microbiome alpha- and beta-diversity according to energy-adjusted quartiles of refined grain intake in the FAMiLI. **A,** Boxplot of Shannon diversity index by refined grain quartiles. **B,** Principal coordinate analysis of the JSD by refined grain quartiles. **C,** Volcano plots of differentially abundant s-OTUs as detected by ANCOM (model adjusted for age, sex, race, BMI, smoking status, alcohol intake, and total energy) between quartile 4 (Q4) and quartile 1 (Q1) of refined grain intake. The *x*-axis represents the difference in mean centered log ratio (clr)-transformed abundance between Q4 and Q1, and the *y*-axis represents the ANCOM W Statistic. s-OTU points are colored according to level of ANCOM significance, with 0.90 being the highest level and gray points indicating s-OTUs that were not significant.

**FIGURE 3:**
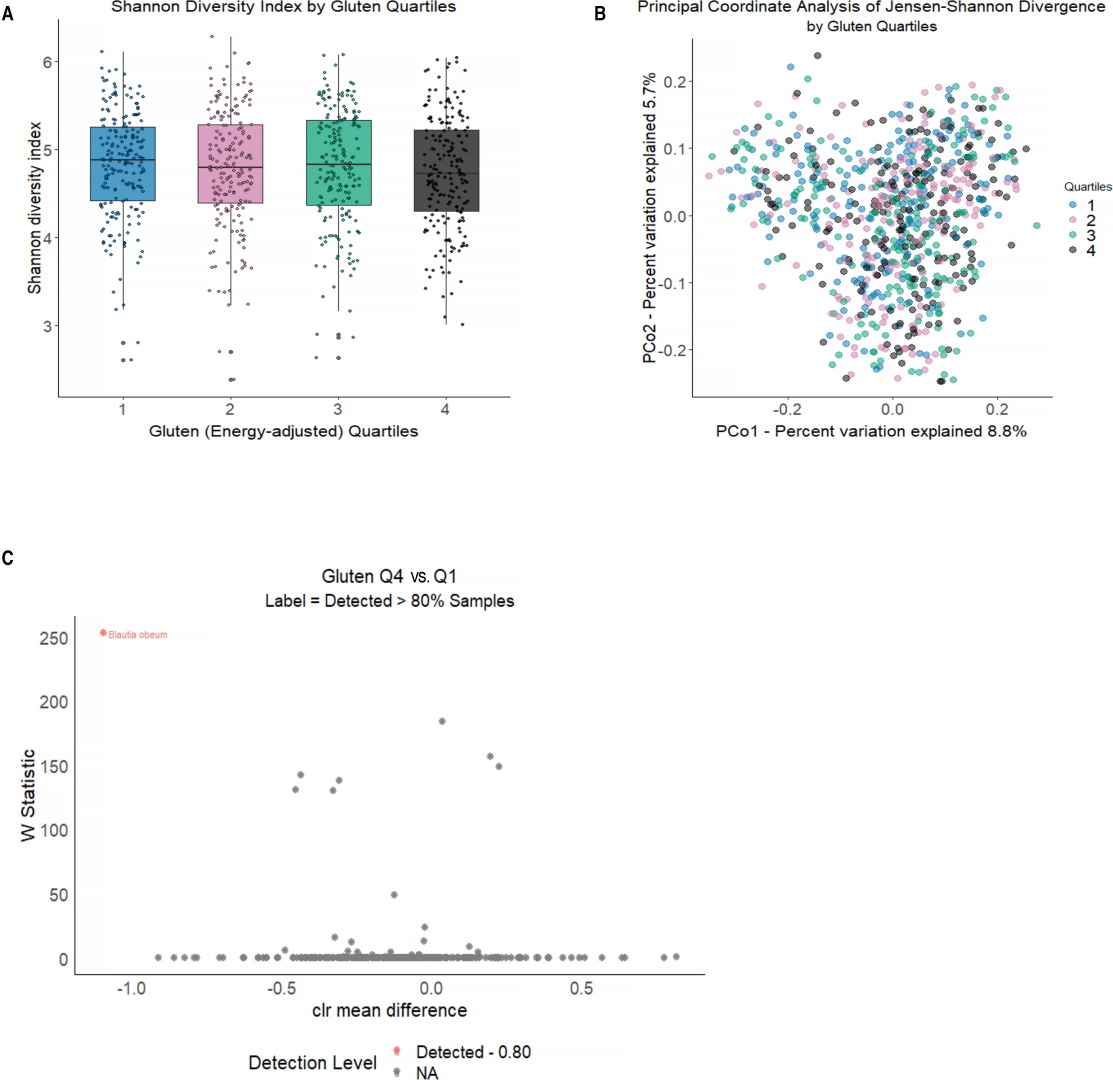
Gut microbiome alpha- and beta-diversity according to energy-adjusted quartiles of gluten intake in the FAMiLI. **A,** Boxplot of Shannon diversity index by gluten quartiles. **B,** Principal coordinate analysis of the JSD by gluten quartiles. **C,** Volcano plots of differentially abundant s-OTUs as detected by ANCOM (model adjusted for age, sex, race, BMI, smoking status, alcohol intake, and total energy) between quartile 4 (Q4) and quartile 1 (Q1) of gluten intake. The *x*-axis represents the difference in mean centered log ratio (clr)-transformed abundance between Q4 and Q1, and the *y*-axis represents the ANCOM W Statistic. s-OTU points are colored according to level of ANCOM significance, with 0.90 being the highest level and gray points indicating s-OTUs that were not significant.

Refined grain consumers were less likely to consume whole grains in their diet (*r* = −0.27). Refined grain consumption was associated with a lower number of observed s-OTUs (richness, *P*_trend_ = 0.04) and lower Shannon diversity index (*P*_trend_ = 0.03; Fig. 2A), but not related to differences in overall microbiome composition (*P*_trend_ = 0.05; Fig. 2B). Greater refined grain consumption was not significantly associated with any specific taxa (Fig. 2C). Most refined grain food items contributed to gluten intake. Thus, refined grain was moderately positively correlated with gluten intake (*r* = 0.46) and weakly negatively correlated with dietary fiber (*r* = −0.18). Higher consumption of gluten was similarly associated with lower Shannon diversity (*P*_trend_ = 0.03), not associated with beta-diversity (JSD *P*_trend_ = 0.19), and not associated with specific gut microbial taxa, other than depletion of s-OTUs of *Blautia obeum* (Fig. 3). Refined grain consumption was associated with lower alpha-diversity after additional adjustment for energy-adjusted dietary fiber and fruit and vegetable intake, although *P* for trend values were no longer statistically significant in most models—(*P*_trend_ for richness = 0.09 and 0.06, respectively; *P*_trend_ for Shannon diversity index = 0.07 and 0.04, respectively), but there remained no differences in overall microbial composition (R^2^ for trend = 0.17% and 0.18%, respectively; *P*_trend_ = 0.11 and 0.08, respectively) or any specific taxa. Additional adjustment for energy-adjusted dietary fiber and fruit and vegetable intake in gluten models did not materially change the results for alpha- (*P*_trend_ for richness = 0.08 and 0.02, respectively; *P*_trend_ for Shannon diversity index = 0.04 and 0.01, respectively) or beta-diversity (R^2^ for trend = 0.16% and 0.15%, respectively; *P*_trend_ = 0.17 and 0.20, respectively) or for taxonomic abundance.

### Sensitivity Analyses

In sensitivity analyses, associations by racial/ethnic group did not significantly differ, but our limited sample size severely limited our ability to examine differences (Supplementary Fig. S2). Similarly, findings remained largely unchanged when we excluded participants who reported a history of type 2 diabetes (energy-adjusted whole grains: *P*_trend_ for richness = 0.84 and *P*_trend_ for Shannon diversity index = 0.96; JSD R^2^ for trend = 0.26% and *P*_trend_ = 0.02) or inflammatory bowel disease (energy-adjusted whole grains: *P*_trend_ for richness = 0.92 and *P*_trend_ for Shannon diversity index = 0.76; JSD R^2^ for trend = 0.21% and *P*_trend_ = 0.02), because grain-based, gluten-containing foods may be avoided with these conditions (Supplementary Figs. S3 and S4). When we included grain-based sweets and dessert items with refined grain (*P*_trend_ for richness = 0.04 and *P*_trend_ for Shannon diversity index = 0.02; JSD R^2^ for trend = 0.20% and *P*_trend_ = 0.03) and gluten intakes (*P*_trend_ for richness = 0.07 and *P*_trend_ for Shannon diversity index = 0.03; JSD R^2^ for trend = 0.15% and *P*_trend_ = 0.18), findings also remained unchanged (Supplementary Figs. S5 and S6).

## Discussion

In this study, we found that whole grain intake influences the overall gut microbial composition, which was largely explained by the higher abundance of *F. prausnitzii*, *B. plebeius*, and Erysipelotrichaceae and lower abundance of *B. uniformis*, Oscillospira, and Rikenellaceae. These bacteria were also associated with higher dietary fiber intakes. We further found that refined grain and gluten consumers have lower Shannon diversity but do not have any significant variation in specific bacterial taxa, even with the inclusion of grain-based sweets and desserts.

In contrast to refined grains, whole grains are a rich source of dietary fiber, nutrients, and various bioactive compounds, such as phytochemicals. These compounds may reduce risk of colorectal cancer and other cancers through various antiproliferative and inhibitory effects against cancer cells (49) that may be at least partially modulated by gut microbiota (50). Through gut microbial fermentation of dietary fibers, the production of the SCFA butyrate is thought to suppress colonic inflammation and carcinogenesis by protecting DNA damage in colonocytes induced by oxidative stress (51), facilitating normal colonocyte growth (52), facilitating assembly of tight junction proteins to maintain the intestinal barrier (53), and inhibiting tumor cell growth (54). However, unlike whole grains, grain fiber was not associated with alpha- or beta-diversity or abundant taxa in this study, which raises the question whether other components of whole grains beyond dietary fiber may be associated with gut microbiota. Whole grains are more complex than refined grains and contain additional nutrients, including unsaturated fatty acids and various phytochemicals, which have unknown associations with the gut microbiome. Future studies that utilize whole genome shotgun sequencing techniques and examine the fecal metabolome will help elucidate food-microbiome relationships.

We found that greater whole grain intake was associated with increased beta-diversity and increased abundance of various SCFA-producing species, including s-OTUs from *F. prausnitzii* and family Erysipelotrichaceae. These findings provide further evidence that whole grain consumption is associated with lower colorectal cancer risk due to an associated healthier gut microbial phenotype. Previous intervention trials of whole grains have yielded mixed results related to *F. prausnitzii* (14–20), which may be partly due to differences in study type (crossover vs. parallel dietary interventions), study length, type of whole grain or dietary fiber, dose, and baseline gut microbiota of different study populations (e.g., healthy vs. obese; ref. 55). Evidence from animal models also suggest that high-fat or Western dietary patterns, which are low in whole grains, may increase abundance of Erysipelotrichaceae (56), but additional studies report higher (57, 58) and lower (59–61) abundances of Erysipelotrichaceae in inflammatory bowel disease and colorectal cancer. This suggests that differences may exist between species. We found that s-OTUs of Erysipelotrichaceae were still enriched with greater whole grain intake after exclusion of participants with self-reported inflammatory bowel disease. Additional studies that utilize whole genome shotgun sequencing are warranted to clarify the relationship between whole grains, SCFA-producing species, and risk of inflammatory bowel disease and colorectal cancer.

Conversely, greater whole grain consumption was associated with lower abundance of s-OTUs of *B. uniformis* and family Rikenellaceae from phylum Bacteroidetes. Although these propionate-producing microbes have not been associated with whole grain or dietary fiber consumption, evidence suggests potential associations with animal protein and dietary fat consumption (62–64). In our study, participants with the highest whole grain intakes consumed less total fat and processed meat and more fruits and vegetables, suggesting that *B. uniformis* and Rikenellaceae may be associated with dietary patterns that are less healthy and commonly low in whole grains and dietary fiber (65).

Higher whole grain intake was associated with higher abundance of s-OTUs of *B. plebeius*, also from phylum Bacteroidetes, but the significance of this finding is uncertain. Although *B. plebeius* was previously reported to have decreased among participants who consumed a 12-week high-fiber rye bread intervention (66), it was also identified to contain an enzyme capable of digesting nori seaweed among native Japanese populations (67). This evidence may at least partially explain our finding because participants with the highest whole grain intake were more likely to be of Korean descent; however, seaweed-containing food items were not included on the original FFQ and therefore, were unable to be assessed.

Current evidence on the relationship between gluten and gut microbiota in healthy adult populations is limited. To our knowledge, three dietary intervention trials of gluten-free (11, 12) or low-gluten (13) diets were conducted in adult non-celiac disease populations. Among 10 Spanish adults ages 23–40 years maintained on a gluten-free diet for 1 month, statistically significant decreases in Bifidobacterium and Lactobacillus and increases in *Escherichia coli* and Enterobacteriaceae compared with baseline were observed (11). A second trial of 21 Dutch men and women ages 16–61 years reported alterations in eight taxa, with the greatest decrease in family Veillonellaceae, after following a 4-week gluten-free diet (12). A third crossover trial of 54 Danish adults ages 22–65 years reported that an 8-week low-gluten diet altered abundance of 14 bacterial species in comparison with a high-gluten diet of equal dietary fiber content, including decreased abundance of four Bifidobacterium species (13). Given that Bifidobacterium and Veillonellaceae species mediate or are directly involved in SCFA production, respectively (68, 69), this evidence suggests that gluten-free dietary patterns may cause undesirable shifts in gut microbial composition. We did not observe similar findings as previous studies, but we did observe a depletion of s-OTUs of *B. obeum* with gluten intake. This, along with our finding that s-OTUs of *B. producta* were enriched with higher whole grain intake, may reflect the association of Blautia genus with visceral fat accumulation and obesity (70). This finding suggests that greater consumption of whole grains, rather than refined grains, which was correlated with gluten intake, may be associated with enrichment of Blautia species. However, additional studies are needed to understand the significance of different Blautia species and to understand whether gluten has an undesirable effect on gut microbial composition in healthy populations.

Additional components of diet, beyond whole grains and dietary fiber, may influence gut microbial composition, such as dietary fat (71) and fruits and vegetables (72), which correlated with whole grain consumption in this population. Other dietary components, such as artificial sweeteners (73), were not assessed in this study. High whole grain consumption may also be indicative of healthier dietary patterns. Dietary fiber is a major component of healthy dietary patterns, and intake was associated with similar microbial taxa as whole grain intake in this study. Grain fiber was not associated with these taxa, suggesting that whole grain components, other than fiber, may influence gut microbiota. Adjustment for fruit and vegetable intake in models of whole grain and dietary fiber did not change associations with microbial taxa, suggesting that fruit and vegetable intake was not responsible for the gut microbial composition associations observed. Similarly, the addition of grain-based sweets and desserts in refined grain and gluten models did not change associations, suggesting that these foods, which are often low in dietary fiber and high in sugar and/or saturated fat, were also not associated with gut microbial composition. Additional studies are needed to elucidate the associations between individual foods and nutrients, as well as overall dietary patterns, on the gut microbiome.

Strengths of this study include a large study population with collection of stool samples and the assessment of habitual, rather than shorter-term, dietary intakes using the FFQ. This allowed us to examine a wide range of whole and refined grain, dietary fiber, and gluten intakes in relation to gut microbial composition. A limitation of this study was the limited sample size when stratifying by race/ethnicity, likely hindering our ability to observe significant differences between groups. Furthermore, Chinese- and Korean-specific grain foods were not available for analysis, which may have contributed to measurement error of the exposures. Because the DQX and DHQ have not been validated in Chinese-American or Korean-American populations (27), we acknowledge that consumption of certain cultural foods may have been underestimated. There is very limited data on dietary patterns of the Korean American population, but commonly consumed Korean foods include kimchi, rice, garlic, green onions, Korean soups and stews, and various Korean condiments (74). Although none of these foods are considered whole grains, kimchi and other vegetables may contribute to vegetable and dietary fiber intake. Thus, underestimation of vegetable dietary fiber intake by the DQX may have contributed to residual confounding. Similarly, refined grain consumption may have been underestimated among Korean American participants, which may have contributed to measurement error and attenuation of the refined grain results. However, rice consumption in the native Korean diet has steadily declined (75) so it is unclear whether or to what degree rice consumption was underestimated by the DQX in this study. Acculturation based on length of residence in the United States may also influence dietary patterns of Korean Americans to be more “Americanized” versus “Korean,” but this information was unavailable in this study. Additional studies are needed to validate the DQX and DHQ among Korean American and other Asian American subgroups. Additional limitations of this study include the cross-sectional design, which did not allow us to examine within-person changes in grain and gluten intakes, gut microbiota, and other lifestyle factors over time, as well as any potential health-related consequences of long-term intake of grain- and gluten-containing foods and related changes in the gut microbiome. With continued enrollment, future analyses in the FAMiLI cohort will have greater statistical power to investigate associations by racial/ethnic group and will be able to examine longitudinal changes in dietary intakes and gut microbiome as repeated stool samples and dietary intakes are collected over time.

In summary, our findings from this cross-sectional study of U.S. adults suggest that higher refined grain and gluten consumption may be associated with lower gut microbial alpha-diversity, and that higher whole grain consumption may be associated with altered gut microbial composition. Although these findings suggest that whole grains, independent of their grain fiber and gluten content, may contribute to a healthier gut microbial profile, additional studies are needed to confirm our findings and provide additional evidence as to how these dietary exposures influence gut microbiota and subsequent risk for chronic diseases, including colorectal cancer.

## Supplementary Material

Supplemental Figures S1-S6 S1. Gut microbiome alpha- and beta-diversity according to energy-adjusted quartiles of dietary fiber intake in the Food and Microbiome Longitudinal Investigation (FAMiLI).
S2. Gut microbiome alpha- and beta-diversity according to energy-adjusted quartiles of whole grain intake in the Food and Microbiome Longitudinal Investigation (FAMiLI), stratified by race/ethnicity.
S3. Gut microbiome alpha- and beta-diversity according to energy-adjusted quartiles of whole grain intake in the Food and Microbiome Longitudinal Investigation (FAMiLI).
S4. Gut microbiome alpha- and beta-diversity according to energy-adjusted quartiles of whole grain intake in the Food and Microbiome Longitudinal Investigation (FAMiLI), excluding participants with self-reported inflammatory bowel disease (N=21) at enrollment.
S5. Gut microbiome alpha- and beta-diversity according to energy-adjusted quartiles of refined grain (with sweets/desserts) intake in the Food and Microbiome Longitudinal Investigation (FAMiLI).
S6. Gut microbiome alpha- and beta-diversity according to energy-adjusted quartiles of gluten (with sweets/desserts) intake in the Food and Microbiome Longitudinal Investigation (FAMiLI).
