# Supplementary material for: Grain, Gluten, and Dietary Fiber Intake Influence Gut Microbial Diversity: Data from the Food and Microbiome Longitudinal Investigation
Source: Cancer Res Commun. 2023 Jan 11;3(1):43–53. doi: 10.1158/2767-9764.CRC-22-0154 (PMC10035461; doi:10.1158/2767-9764.CRC-22-0154)

Supplemental Figure 1. Gut microbiome alpha- and beta-diversity according to energy-adjusted quartiles of dietary fiber intake in the Food and Microbiome Longitudinal Investigation (FAMiLI). a) Boxplot of Shannon Diversity Index by dietary fiber quartiles. b) Principal coordinate analysis of the Jensen-Shannon Divergence (JSD) by dietary fiber quartiles. c) Volcano plots of differentially abundant s-OTUs as detected by ANCOM (model adjusted for age, sex, race, BMI, smoking status, alcohol intake, and total energy) between quartile 4 (Q4) and quartile 1 (Q1) of dietary fiber intake. The x-axis represents the difference in mean centered log ratio (clr)-transformed abundance between Q4 and Q1, and the y-axis represents the ANCOM W Statistic. s-OTU points are colored according to level of ANCOM significance, with 0.90 being the highest level and grey points indicating s-OTUs that were not significant.

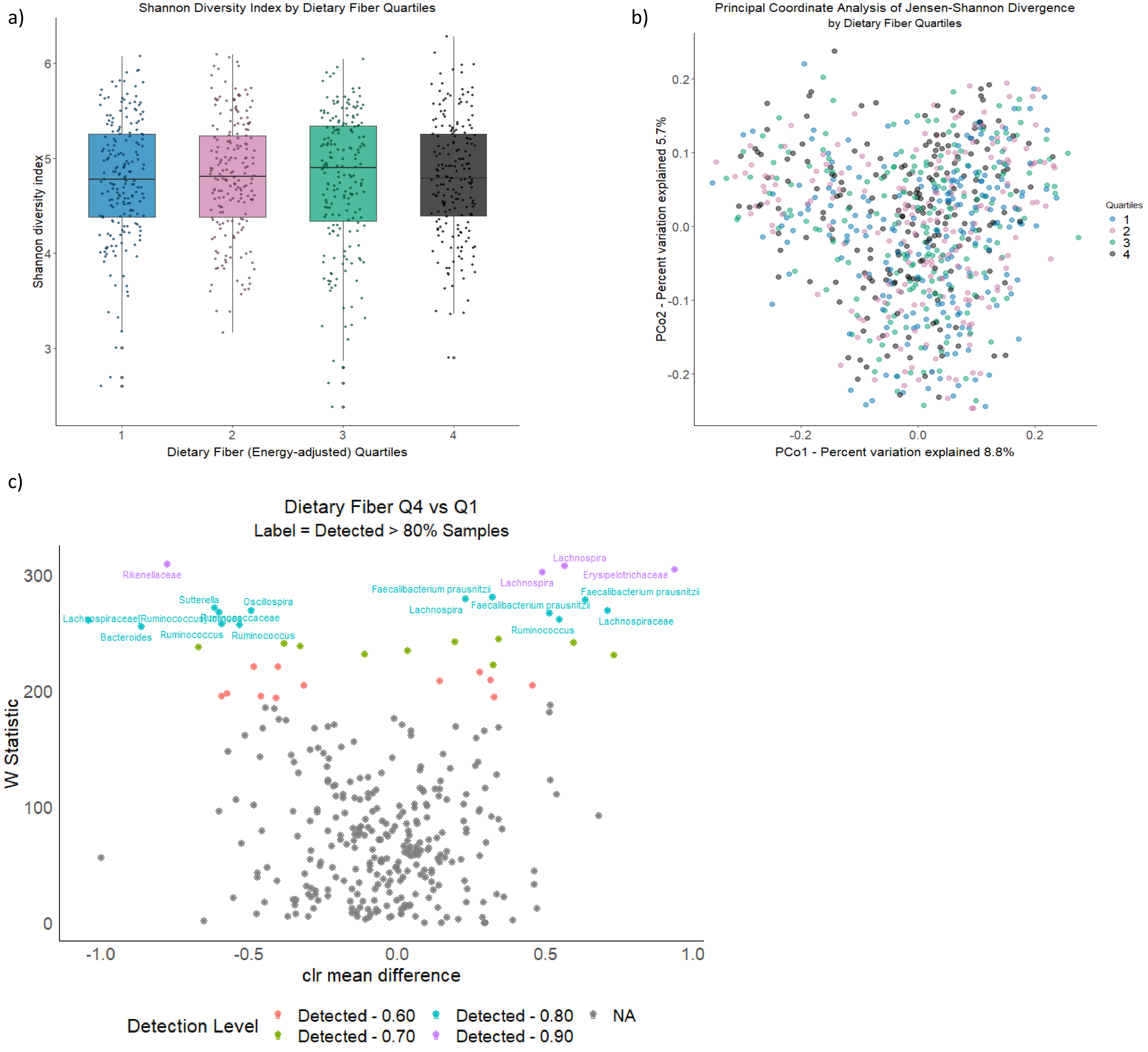

Supplemental Figure 2. Gut microbiome alpha- and beta-diversity according to energy-adjusted quartiles of whole grain intake in the Food and Microbiome Longitudinal Investigation (FAMiLI), stratified by race/ethnicity (White (N=297), Black (N=84), Hispanic (N=121), Korean (N=244), Other Asian (N=25), Other (N=8)). a) Boxplot of richness by whole grain quartiles, stratified by race/ethnicity. b) Boxplot of Shannon Diversity Index by whole grain quartiles, stratified by race/ethnicity. c) Principal coordinate analysis of the Jensen-Shannon Divergence (JSD) by whole grain quartiles for: i) White, ii) Black, iii) Hispanic, iv) Korean, and v) Other Asian participants.

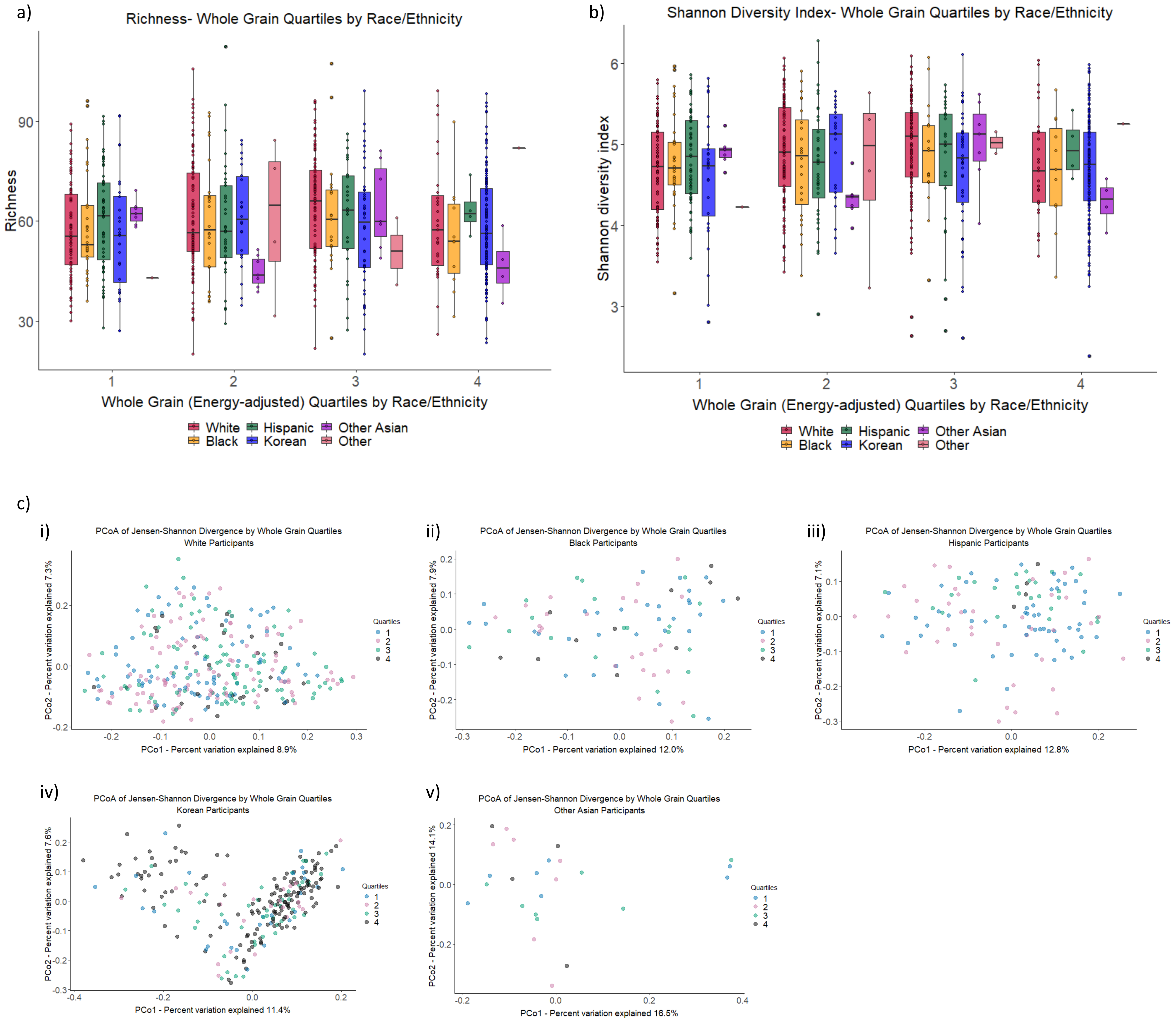

Supplemental Figure 3. Gut microbiome alpha- and beta-diversity according to energy-adjusted quartiles of whole grain intake in the Food and Microbiome Longitudinal Investigation (FAMiLI), excluding participants with self-reported type 2 diabetes (N=130) at enrollment. a) Boxplot of Shannon Diversity Index by whole grain quartiles. b) Principal coordinate analysis of the Jensen-Shannon Divergence (JSD) by whole grain quartiles. c) Volcano plots of differentially abundant s-OTUs as detected by ANCOM (model adjusted for age, sex, race, BMI, smoking status, alcohol intake, and total energy) between quartile 4 (Q4) and quartile 1 (Q1) of whole grain intake. The x-axis represents the difference in mean centered log ratio (clr)-transformed abundance between Q4 and Q1, and the y-axis represents the ANCOM W Statistic. s-OTU points are colored according to level of ANCOM significance, with 0.90 being the highest level and grey points indicating s-OTUs that were not significant.

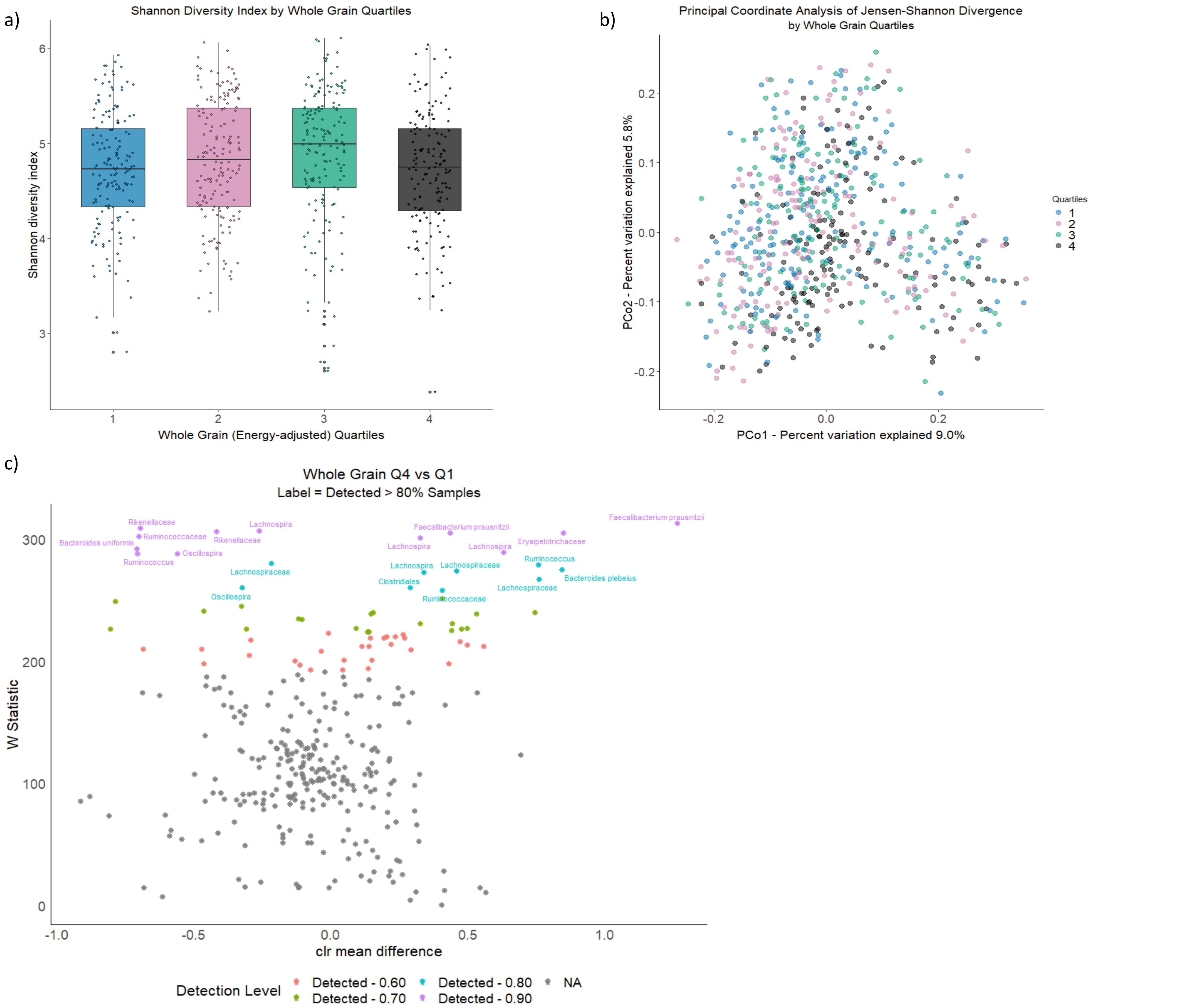

Supplemental Figure 4. Gut microbiome alpha- and beta-diversity according to energy-adjusted quartiles of whole grain intake in the Food and Microbiome Longitudinal Investigation (FAMiLI), excluding participants with self-reported inflammatory bowel disease (N=21) at enrollment. a) Boxplot of Shannon Diversity Index by whole grain quartiles. b) Principal coordinate analysis of the Jensen-Shannon Divergence (JSD) by whole grain quartiles. c) Volcano plots of differentially abundant s-OTUs as detected by ANCOM (model adjusted for age, sex, race, BMI, smoking status, alcohol intake, and total energy) between quartile 4 (Q4) and quartile 1 (Q1) of whole grain intake. The x-axis represents the difference in mean centered log ratio (clr)-transformed abundance between Q4 and Q1, and the y-axis represents the ANCOM W Statistic. s-OTU points are colored according to level of ANCOM significance, with 0.90 being the highest level and grey points indicating s-OTUs that were not significant.

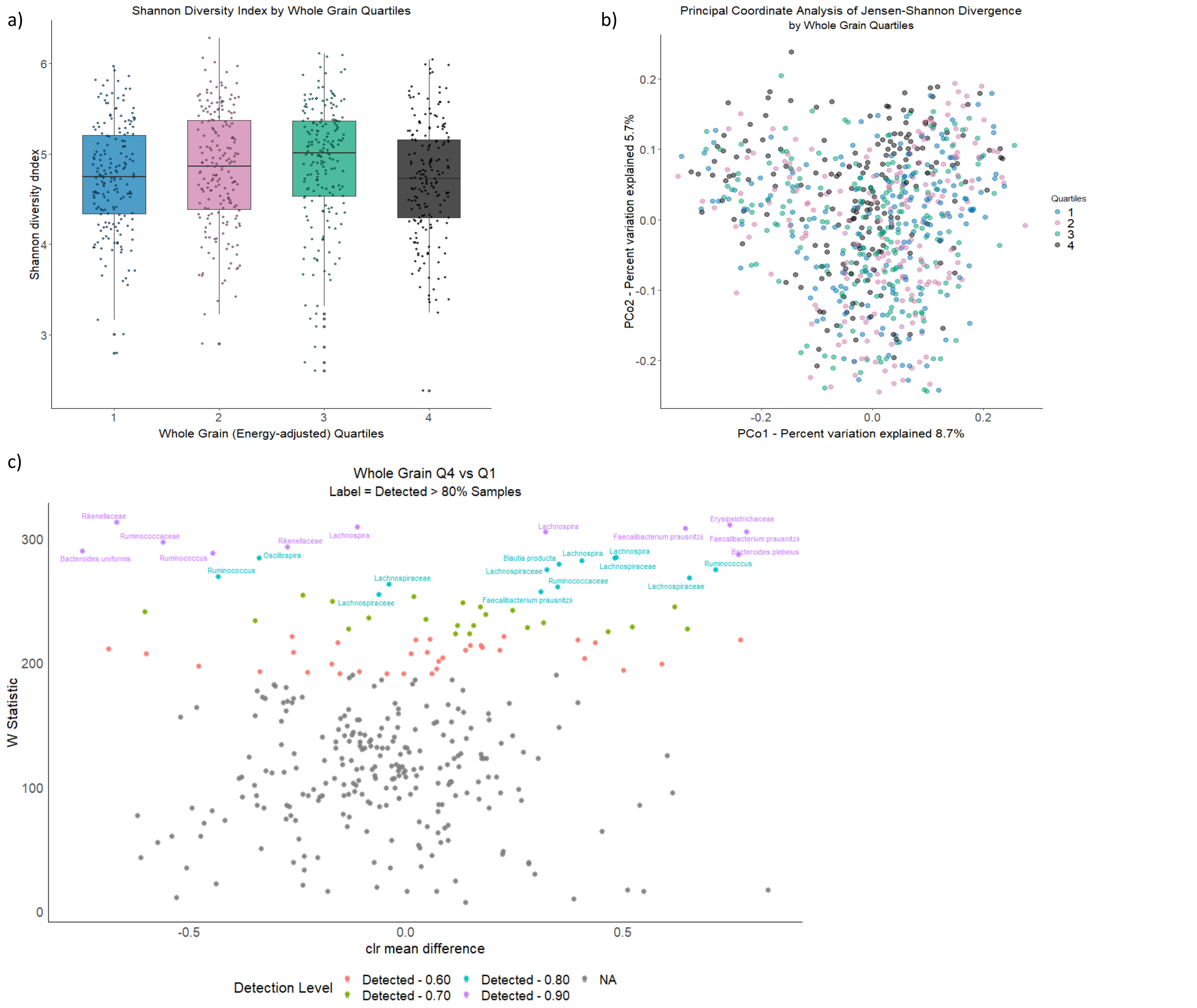

Supplemental Figure 5. Gut microbiome alpha- and beta-diversity according to energy-adjusted quartiles of refined grain (with sweets/desserts) intake in the Food and Microbiome Longitudinal Investigation (FAMiLI). a) Boxplot of Shannon Diversity Index by refined grain (with sweets/desserts) quartiles. b) Principal coordinate analysis of the Jensen-Shannon Divergence (JSD) by refined grain (with sweets/desserts) quartiles. c) Volcano plots of differentially abundant s-OTUs as detected by ANCOM (model adjusted for age, sex, race, BMI, smoking status, alcohol intake, and total energy) between quartile 4 (Q4) and quartile 1 (Q1) of refined grain (with sweets/desserts) intake. The x-axis represents the difference in mean centered log ratio (clr)-transformed abundance between Q4 and Q1, and the y-axis represents the ANCOM W Statistic. s-OTU points are colored according to level of ANCOM significance, with 0.90 being the highest level and grey points indicating s-OTUs that were not significant.

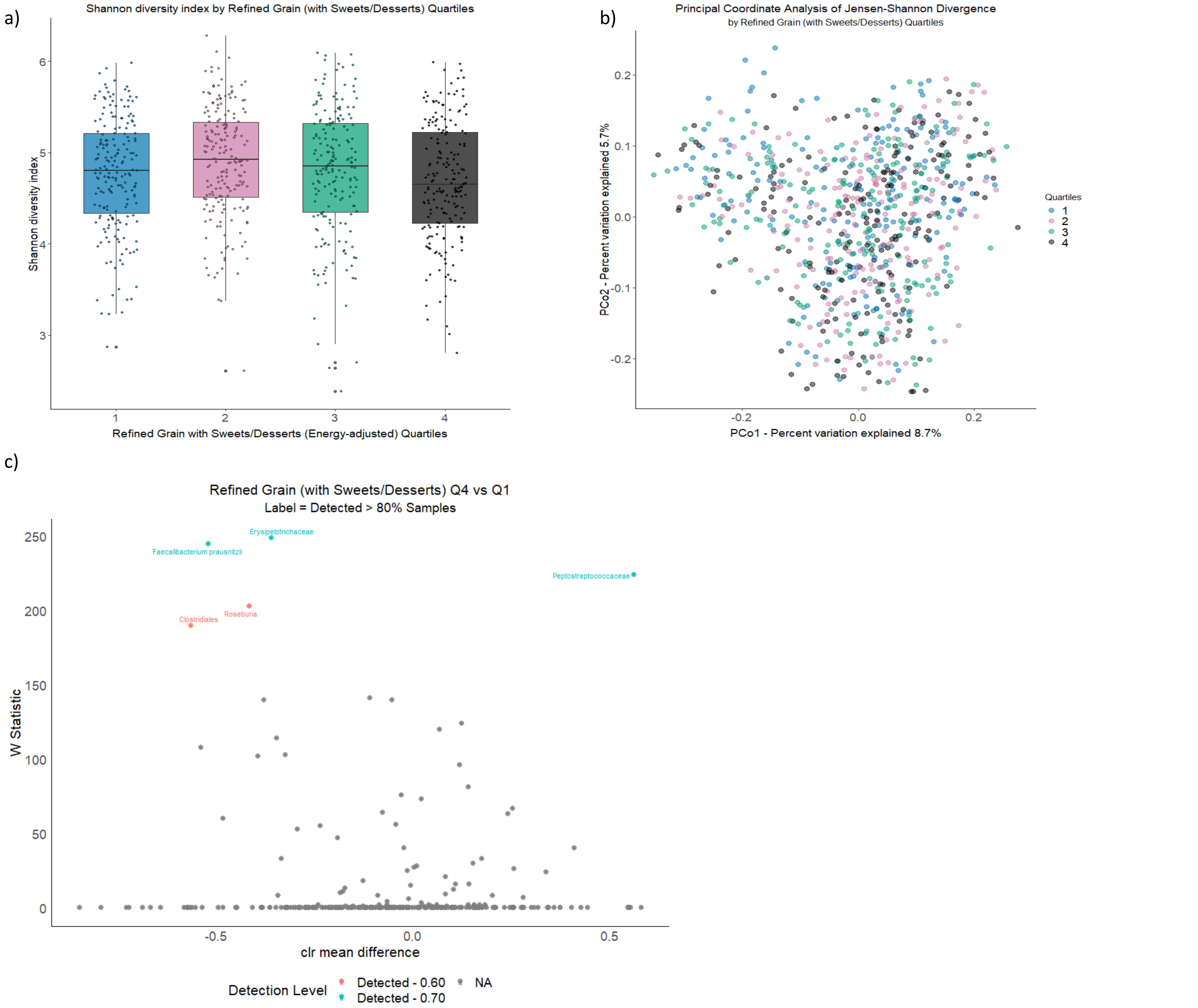

Supplemental Figure 6. Gut microbiome alpha- and beta-diversity according to energy-adjusted quartiles of gluten (with sweets/desserts) intake in the Food and Microbiome Longitudinal Investigation (FAMiLI). a) Boxplot of Shannon Diversity Index by gluten (with sweets/desserts) quartiles. b) Principal coordinate analysis of the Jensen-Shannon Divergence (JSD) by gluten (with sweets/desserts) quartiles. c) Volcano plots of differentially abundant s-OTUs as detected by ANCOM (model adjusted for age, sex, race, BMI, smoking status, alcohol intake, and total energy) between quartile 4 (Q4) and quartile 1 (Q1) of gluten (with sweets/desserts) intake. The x-axis represents the difference in mean centered log ratio (clr)-transformed abundance between Q4 and Q1, and the y-axis represents the ANCOM W Statistic. s-OTU points are colored according to level of ANCOM significance, with 0.90 being the highest level and grey points indicating s-OTUs that were not significant.

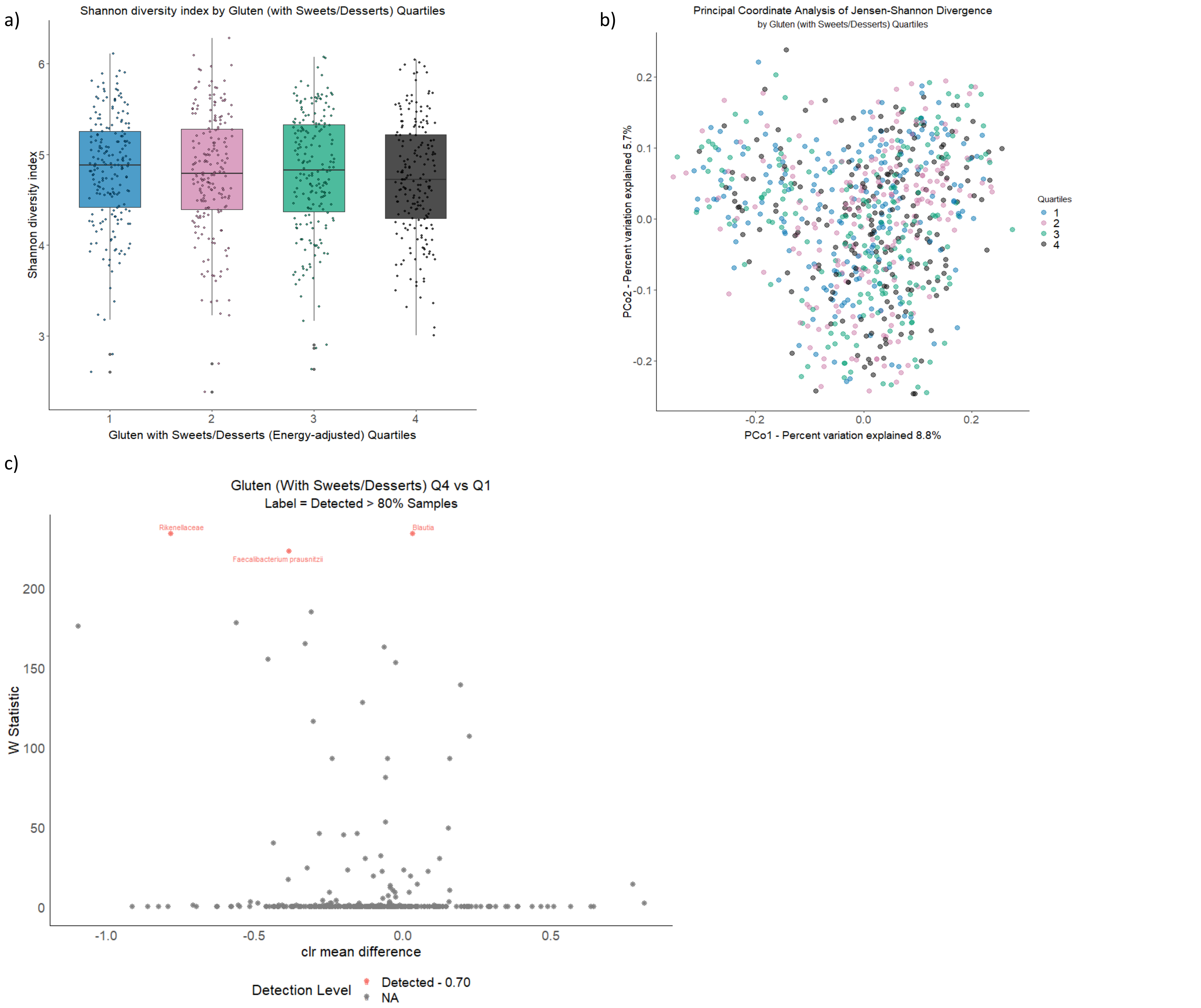

Supplement: Supplemental Figures S1-S6 — S1. Gut microbiome alpha- and beta-diversity according to energy-adjusted quartiles of dietary fiber intake in the Food and Microbiome Longitudinal Investigation (FAMiLI). S2. Gut microbiome alpha- and beta-diversity according to energy-adjusted quartiles of whole grain intake in the Food and Microbiome Longitudinal Investigation (FAMiLI), stratified by race/ethnicity. S3. Gut microbiome alpha- and beta-diversity according to energy-adjusted quartiles of whole grain intake in the Food and Microbiome Longitudinal Investigation (FAMiLI). S4. Gut microbiome alpha- and beta-diversity according to energy-adjusted quartiles of whole grain intake in the Food and Microbiome Longitudinal Investigation (FAMiLI), excluding participants with self-reported inflammatory bowel disease (N=21) at enrollment. S5. Gut microbiome alpha- and beta-diversity according to energy-adjusted quartiles of refined grain (with sweets/desserts) intake in the Food and Microbiome Longitudinal Investigation (FAMiLI). S6. Gut microbiome alpha- and beta-diversity according to energy-adjusted quartiles of gluten (with sweets/desserts) intake in the Food and Microbiome Longitudinal Investigation (FAMiLI). [file crc-22-0154-s01.pdf]
